# Supplementary material for: Th17-associated cytokines IL-17 and IL-23 in inflamed skin of Darier disease patients as potential therapeutic targets
Source: Nat Commun. 2023 Nov 17;14:7470. doi: 10.1038/s41467-023-43210-5 (PMC10656568; doi:10.1038/s41467-023-43210-5)
Supplement: Supplementary file 1 — Supplementary Information [file 41467_2023_43210_MOESM1_ESM.pdf]

Supplementary Information

Contents

Supplementary Tables 2

Supplementary Table 1: Enriched pathways in keratinocytes of DD patients vs. Healthy Controls as determined by Gene Set Enrichment Analysis of scRNA-seq data. 2

Supplementary Table 2: Enriched Biocarta pathway gene sets in Darier Disease patients vs. Healthy Controls as determined by Gene Set Enrichment Analysis of NanoString data. 3

Supplementary Table 3: Enriched Biocarta pathway gene sets in Psoriasis patients vs. Healthy Controls as determined by Gene Set Enrichment Analysis of NanoString data. 4

Supplementary Figures 5

Supplementary Fig 1: Disease phenotype/severity 5

Supplementary Fig 2: scRNA-seq characterization of cell types 6

Supplementary Fig 3: Volcano plot of differentially expressed genes in keratinocytes of DD (N=4) patients vs HC (N=3) 7

Supplementary Fig 4: Cytokine mRNA expression of each single patient 8

Supplementary Fig 5: Isotype controls of multi-color IF images for IL-17A and IL-23A stainings (both Rabbit IgG) 9

Supplementary Fig 6: Immunofluorescence stainings of IL-17A and CD3 10

Supplementary Fig 7: Strategy for gating IL-17A^{+} CD4^{+} cTrm in PBMCs. 11

Supplementary Fig 8: Clinical pictures during treatment of DD PAT1, PAT2 and PAT9 12

Supplementary Fig 9: Improved itch and quality of life of DD patients treated with biologics. 13

Supplementary Fig 10: Strategy for gating IL-17A^{+} CD4^{+} T cells in single cell suspension from human skin. 14

Supplementary Methods 15

Immunofluorescence. 15

Supplementary Tables

Supplementary Table 1: Enriched pathways in keratinocytes of DD patients vs. Healthy Controls as determined by Gene Set Enrichment Analysis of scRNA-seq data.

Pathway name	Overlap	P value	Adjusted p value	database
mTORC1 Signaling	9/200	0.00024	0.00876	MSigDB_Hallmark_2020
p53 Pathway	8/200	0.00113	0.0209	MSigDB_Hallmark_2020
Toll-like receptor binding (GO:0035325)	3/11	0.00017	0.04531	GO_Molecular_Function_2021
calcium ion binding (GO:0005509)	11/348	0.00101	0.06961	GO_Molecular_Function_2021
RNA binding (GO:0003723)	27/1406	0.0013	0.06961	GO_Molecular_Function_2021
icosatetraenoic acid binding (GO:0050543)	2/6	0.00154	0.06961	GO_Molecular_Function_2021
arachidonic acid binding (GO:0050544)	2/6	0.00154	0.06961	GO_Molecular_Function_2021
gap junction channel activity involved in cell communication by electrical coupling (GO:1903763)	2/6	0.00154	0.06961	GO_Molecular_Function_2021
icosanoid binding (GO:0050542)	2/7	0.00214	0.07259	GO_Molecular_Function_2021
insulin-like growth factor II binding (GO:0031995)	2/7	0.00214	0.07259	GO_Molecular_Function_2021
metal ion binding (GO:0046872)	13/517	0.00282	0.08485	GO_Molecular_Function_2021
snoRNA binding (GO:0030515)	3/30	0.00356	0.08928	GO_Molecular_Function_2021
RAGE receptor binding (GO:0050786)	2/9	0.00362	0.08928	GO_Molecular_Function_2021
RB DN.V1 DN	9/126	6.4E-06	0.00099	MSigDB_Oncogenic_Signatures

Supplementary Table 2: Enriched Biocarta pathway gene sets in Darier Disease patients vs. Healthy Controls as determined by Gene Set Enrichment Analysis of NanoString data.

IL-17 signaling pathway is highlighted in bold. FDR, false discovery rate

Biocarta pathway name	No of genes in Nanostring panel	normalized enrichment score	FDR q-value
Cytokines and inflammatory response	21	2.1	0.05
Local acute inflammatory response	15	2.05	0.07
Adhesion and diapedesis of granulocytes	13	2.03	0.074
**IL-17 signaling**	12	1.98	0.124
Cytokine network	17	1.86	0.193
Cytotoxic T lymphocytes	11	1.84	0.196
Regulation of hematopoiesis by cytokines	12	1.81	0.18
NFkB activation by nontypeable Hemophilus influenzae	14	1.75	0.196
T helper cells	10	1.71	0.225
Adhesion and diapedesis of lymphocytes	12	1.71	0.207
T cytotoxic cells	10	1.68	0.219
Free radical induced apoptosis	4	1.66	0.216
Adhesion molecules on lymphocytes	9	1.61	0.217
HIV induced T cell apoptosis	7	1.62	0.221
Co-stimulatory signal during T-cell activation	13	1.6	0.212
Monocytes and its surface molecules	10	1.56	0.226

Supplementary Table 3: Enriched Biocarta pathway gene sets in Psoriasis patients vs. Healthy Controls as determined by Gene Set Enrichment Analysis of NanoString data.

Pathways that are also enriched in Darier Disease patients are highlighted in bold. FDR, false discovery rate

Biocarta pathway name	No of genes in Nanostring panel	normalized enrichment score	FDR q-value
**IL-17 signaling**	12	2.32	0.174
**Cytokine network**	17	2.27	0.174
**Regulation of hematopoiesis by cytokines**	12	2.11	0.174
**NFkB activation by nontypeable Hemophilus influenzae**	14	2.06	0.174
**Cytokines and inflammatory response**	21	1.82	0.174
**Free radical Induced apoptosis**	4	1.82	0.174
IL22 Soluble receptor signaling	9	1.76	0.174
**Adhesion and diapedesis of granulocytes**	13	1.74	0.174
Mechanism of gene regulation by peroxisome proliferators via PPARa	8	1.72	0.174
**Local acute inflammatory response**	15	1.6	0.224
**Adhesion and diapedesis of lymphocytes**	12	1.59	0.22
Antigen processing and presentation	11	1.52	0.231

Supplementary Figures

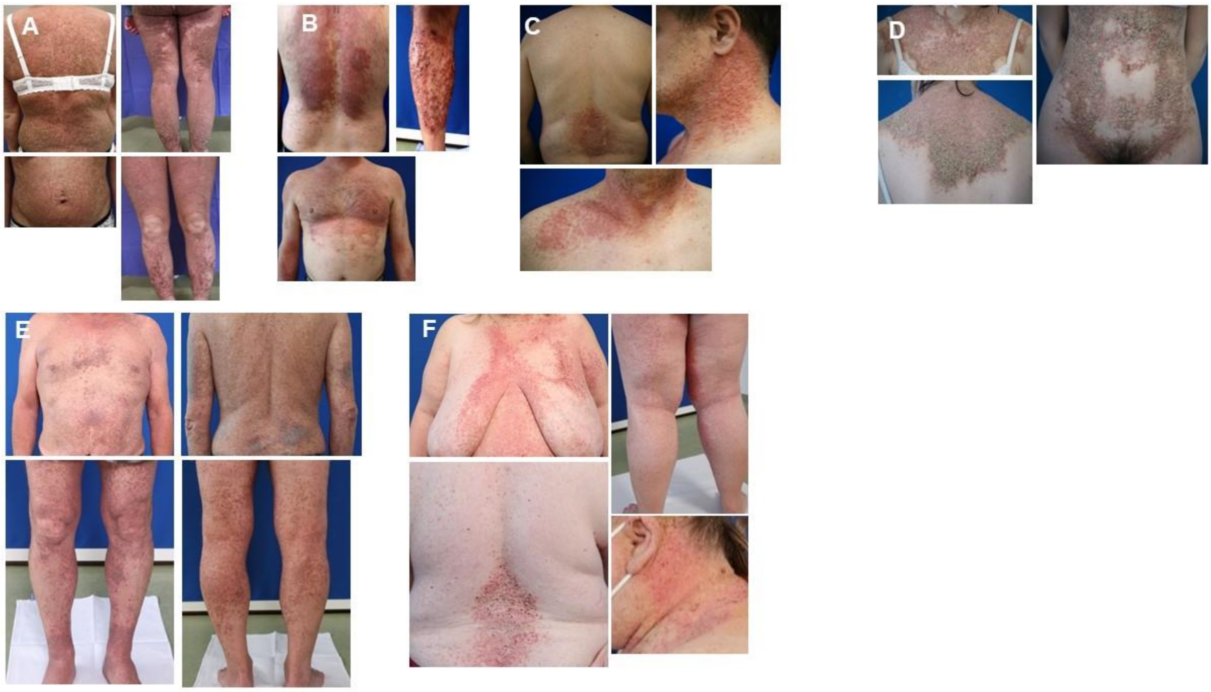

Supplementary Fig 1: Disease phenotype/severity

A PAT1: hyperkeratotic erythematous and brownish papules and plaques on the entire integument, on legs additionally erosions and crusts (severe, >35% BSA affected). **B** PAT2: hyperkeratotic erythematous and brownish papules and plaques on the entire integument, on the legs additionally erosions and crusts (severe, >35% BSA affected). **C** PAT4: hyperkeratotic erythematous and brownish papules and plaques on the upper thorax and neck (moderate, 20% BSA affected). **D** PAT5: hyperkeratotic erythematous and brownish papules and plaques on the entire integument (severe, >35% BSA affected). **E** PAT7 hyperkeratotic erythematous and brownish papules and plaques on the entire integument (severe, >35% BSA affected). **F** PAT9: hyperkeratotic erythematous and brownish papules and plaques on the entire integument (severe, >35% BSA affected). BSA, body surface area

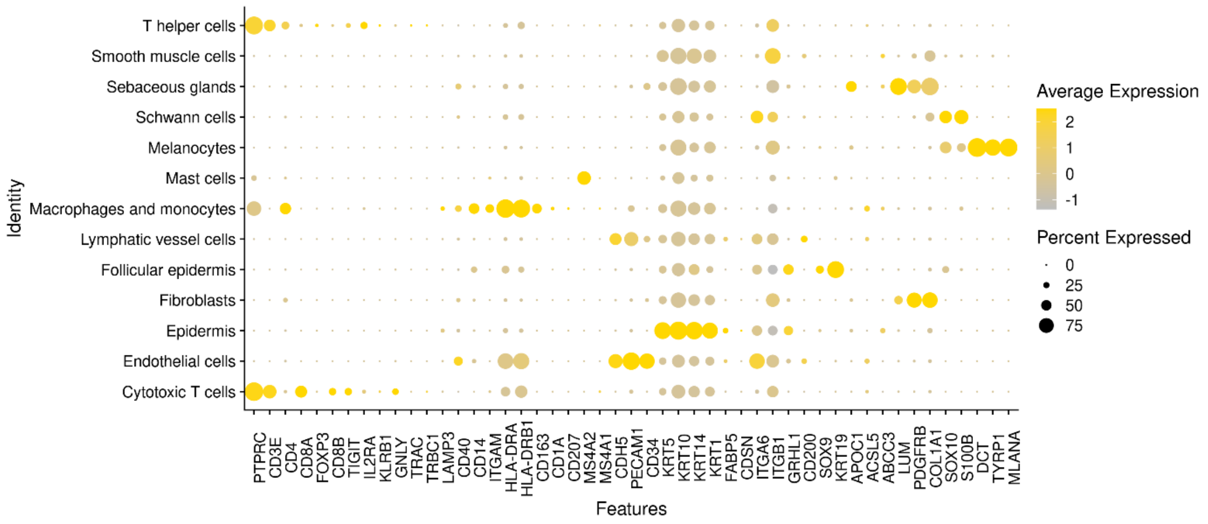

Supplementary Fig 2: scRNA-seq characterization of cell types

Dot plot demonstrating mean log normalized expression of cell type-specific marker genes in integrated scRNAseq data of skin of four DD patients (GSE235255 [https://www.ncbi.nlm.nih.gov/geo/query/acc.cgi?acc=GSE235255]), three Psoriasis patients and three healthy controls (GEO dataset GSE162183 [https://www.ncbi.nlm.nih.gov/geo/query/acc.cgi?acc=GSE162183]). Color scale indicates mean expression, and circle size indicates fraction of cells in a group.

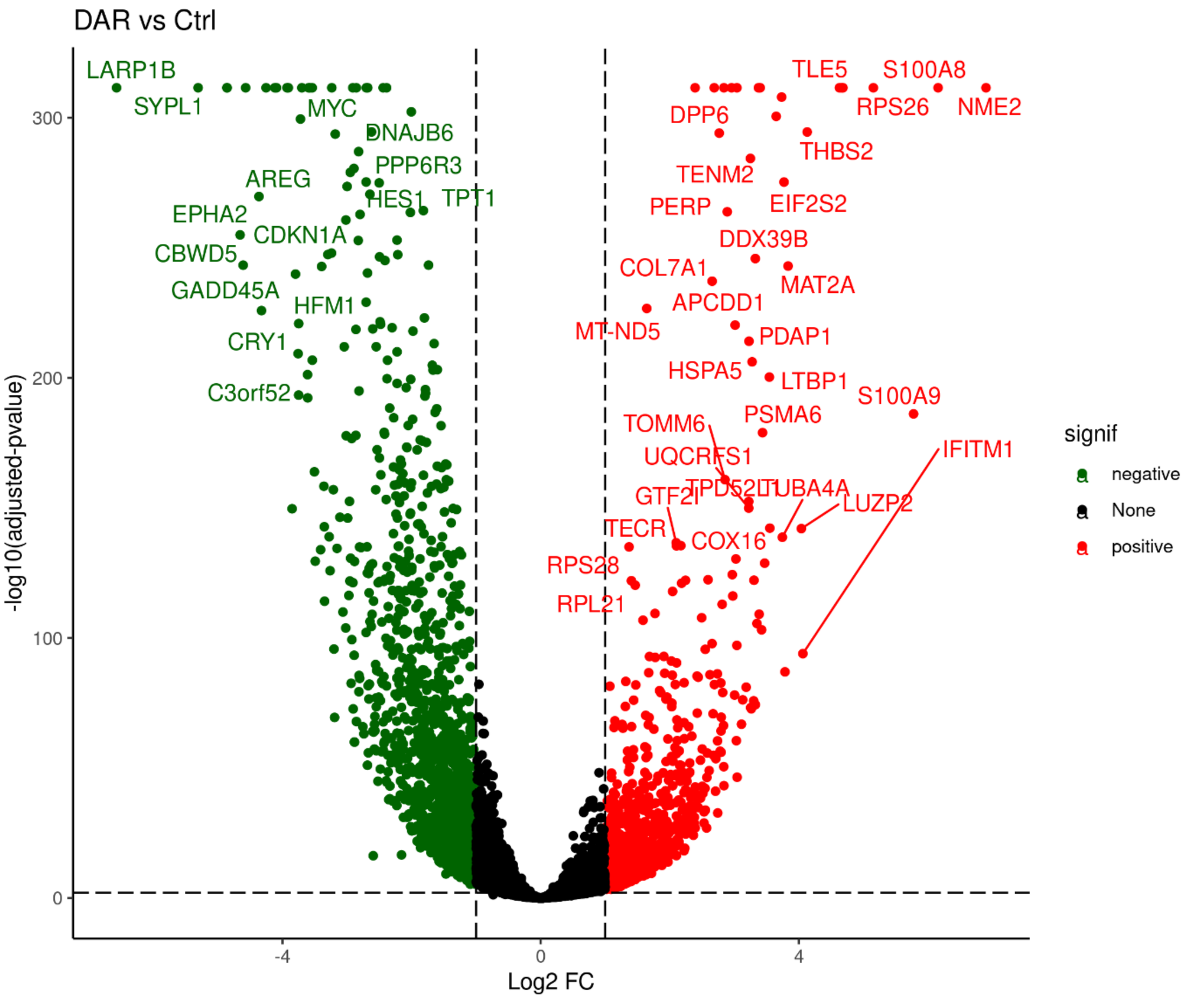

Supplementary Fig 3: Volcano plot of differentially expressed genes in keratinocytes of DD (N=4) patients vs HC (N=3)

scRNA-seq revealed a plethora of DE genes in keratinocytes of DD patients (GSE235255 [https://www.ncbi.nlm.nih.gov/geo/query/acc.cgi?acc=GSE235255]) as compared to healthy control skin (GSE162183, [https://www.ncbi.nlm.nih.gov/geo/query/acc.cgi?acc=GSE162183]).

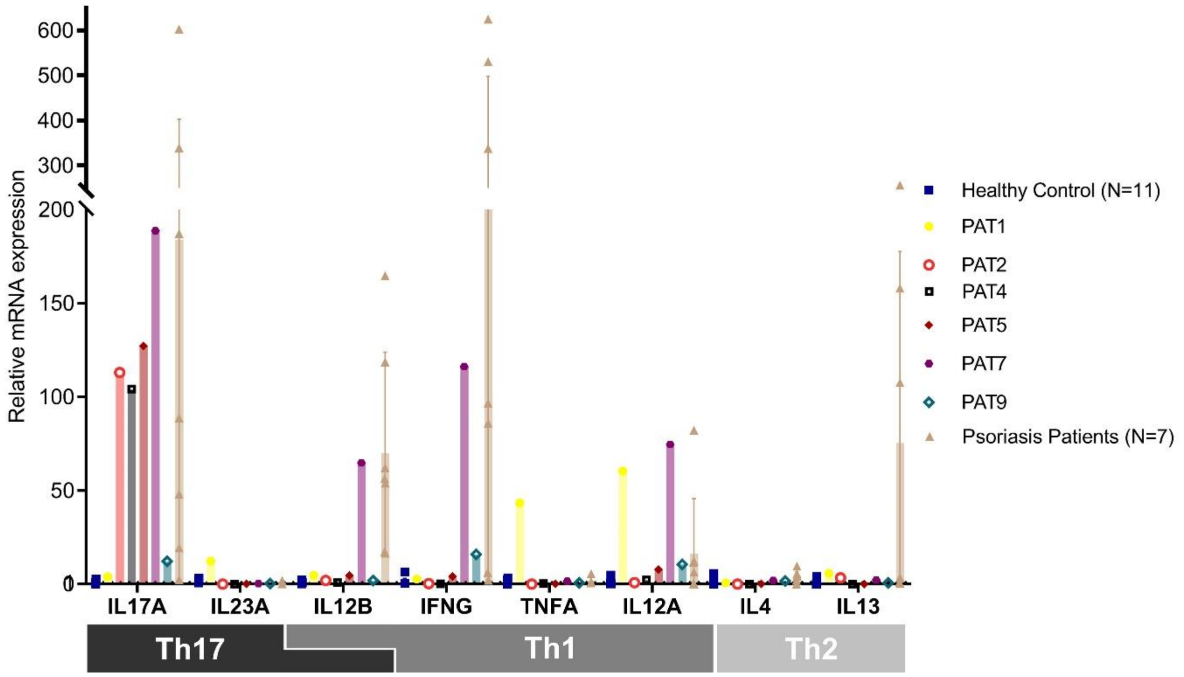

Supplementary Fig 4: Cytokine mRNA expression of each single patient

Cytokine mRNA expression of each single patient was determined by qPCR using probes against the indicated cytokines. Ct values were normalized to housekeeping gene ACTB and healthy controls (N=11). Data points represent means of at least two experimental replicates of individual patients/controls. Bars represent means, error bars represent standard deviations.

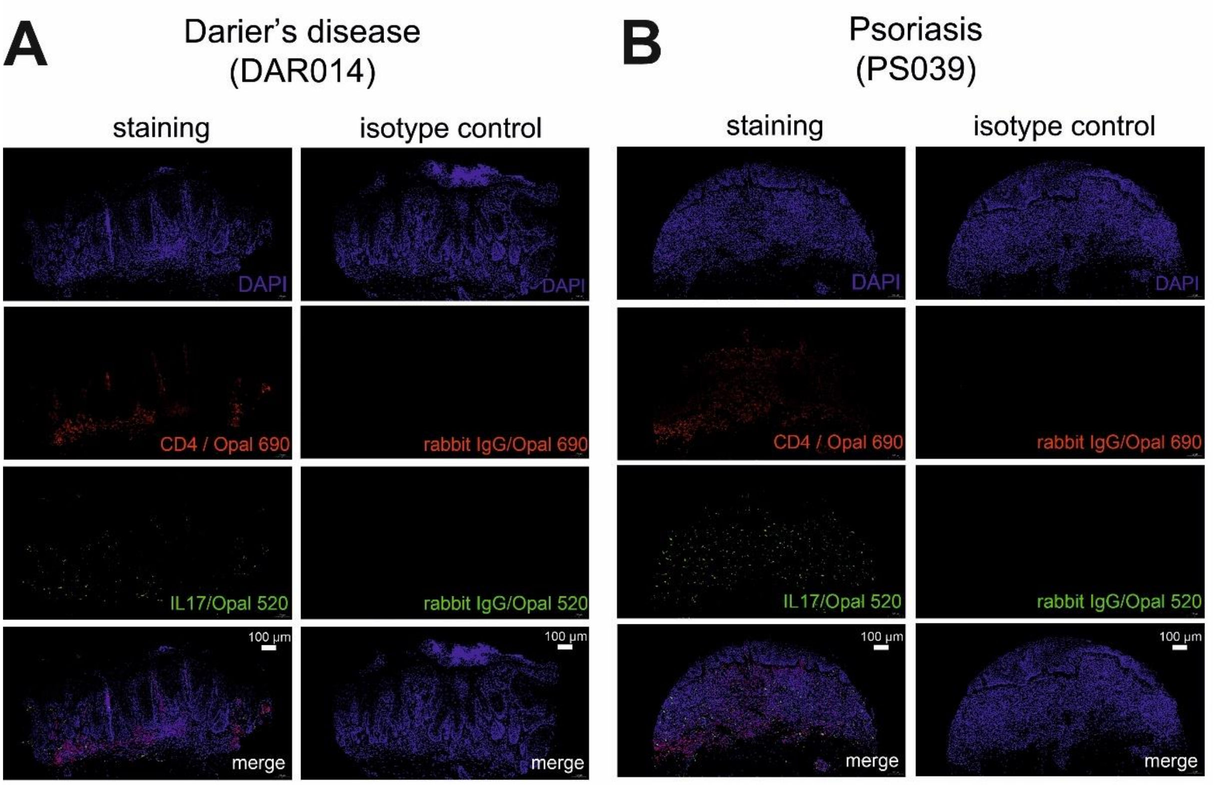

Supplementary Fig 5: Isotype controls of multi-color IF images for IL-17A and IL-23A stainings (both Rabbit IgG)

Isotype controls of OPAL-stained Tissue Microarray sections with seven DD patient, three healthy control and three psoriasis patient cores. **A** Immunofluorescence image of IL-17A$^+$CD4$^+$ and isotype control from one representative DD patient. B Immunofluorescence image of IL-17A$^+$CD4$^+$ and isotype control from one representative psoriasis patient (PSO). CD4 positive T cells in red, IL-17A/IL-23A positive cells in green, DAPI-stained nuclei in blue.

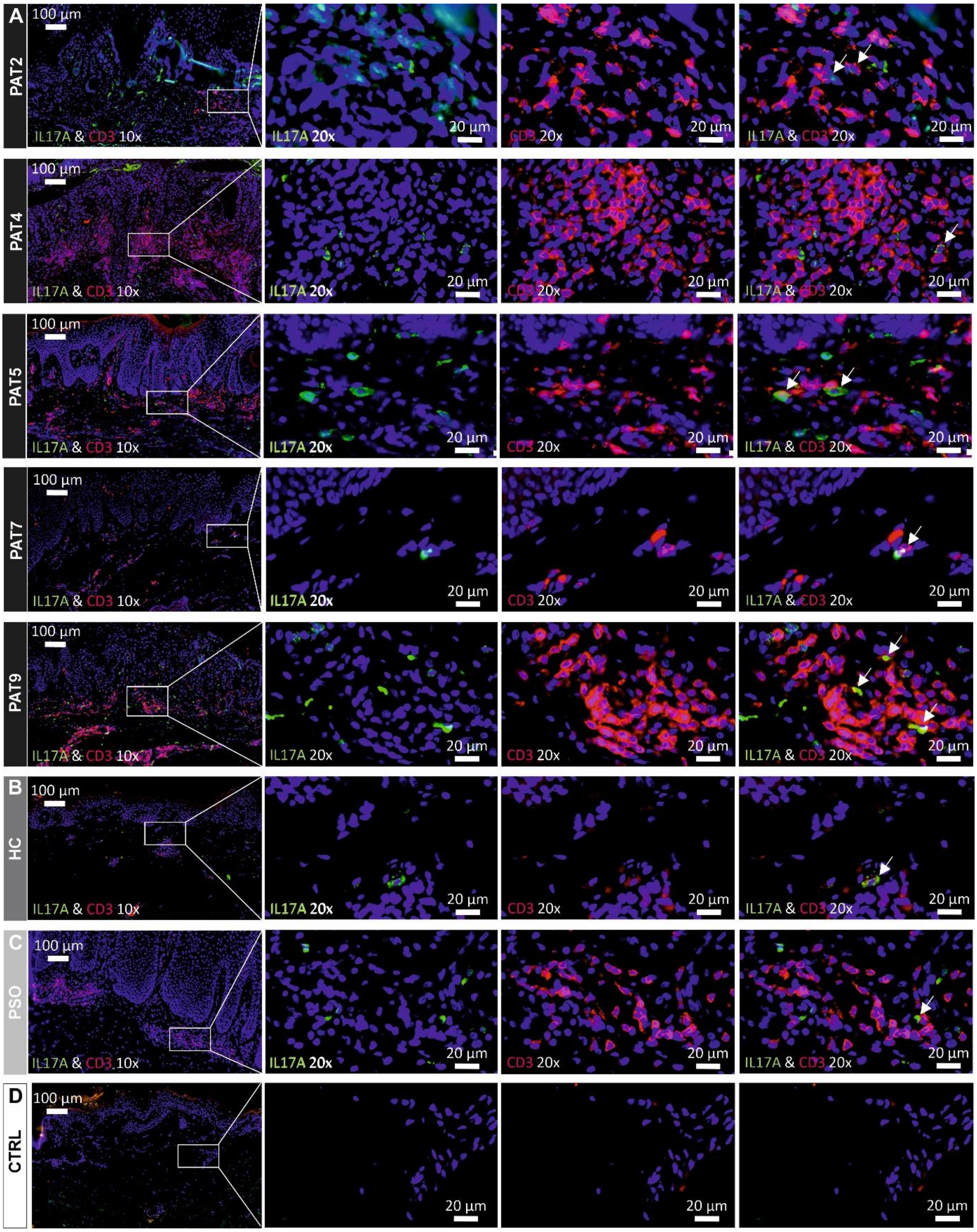

Supplementary Fig 6: Immunofluorescence stainings of IL-17A and CD3

A Skin sections of DD patients (PAT2-9) were stained with antibodies against IL-17A and CD3. **B** Representative immunofluorescence image of IL-17A and CD3 expression in the skin of a healthy control (HC). **C** Representative immunofluorescence image of IL-17A and CD3 expression in the skin of a psoriasis patient (PSO). CD3 positive T cells in red, IL-17A positive cells in green, DAPI-stained nuclei in blue. White arrows indicate IL-17A/CD3 double-positive cells (supposedly Th17/Tc17 cells) in 20x magnified images. **D** Secondary AB control

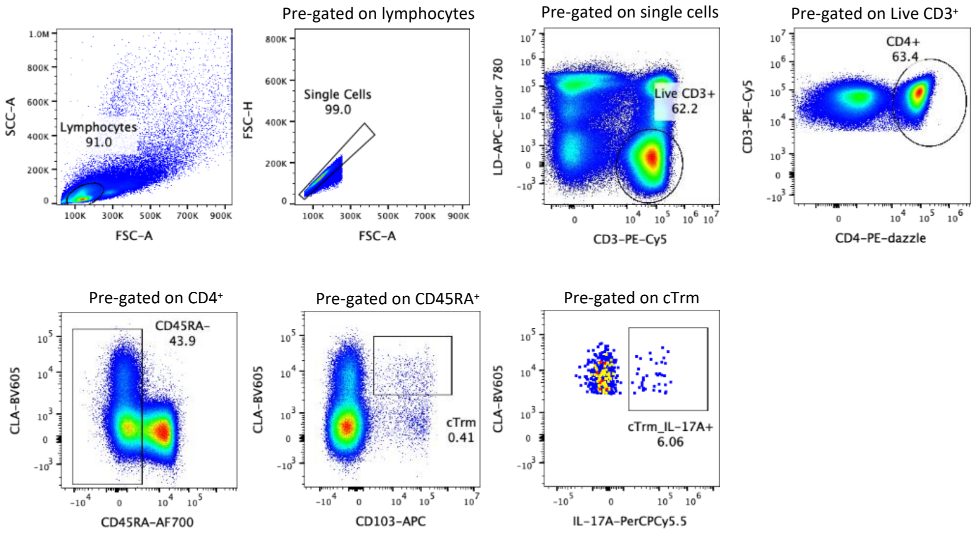

Supplementary Fig 7: Strategy for gating IL-17A^{+} CD4^{+} cTrm in PBMCs.

Lymphocytes were gated using physical parameters; forward scatter - area (FSC-A) and side scatter – area (SCC-A). Doublets were excluded using FSC-A and FSC-H. Further, dead cells were excluded (positive for APC eflour 780 signal) and live CD3^{+} T cell population was gated. Thereafter, CD4^{+} T cells were gated for memory cTrm population as CD45RA^{-}CLA^{+}CD103^{+} and then finally for IL-17A producing population.

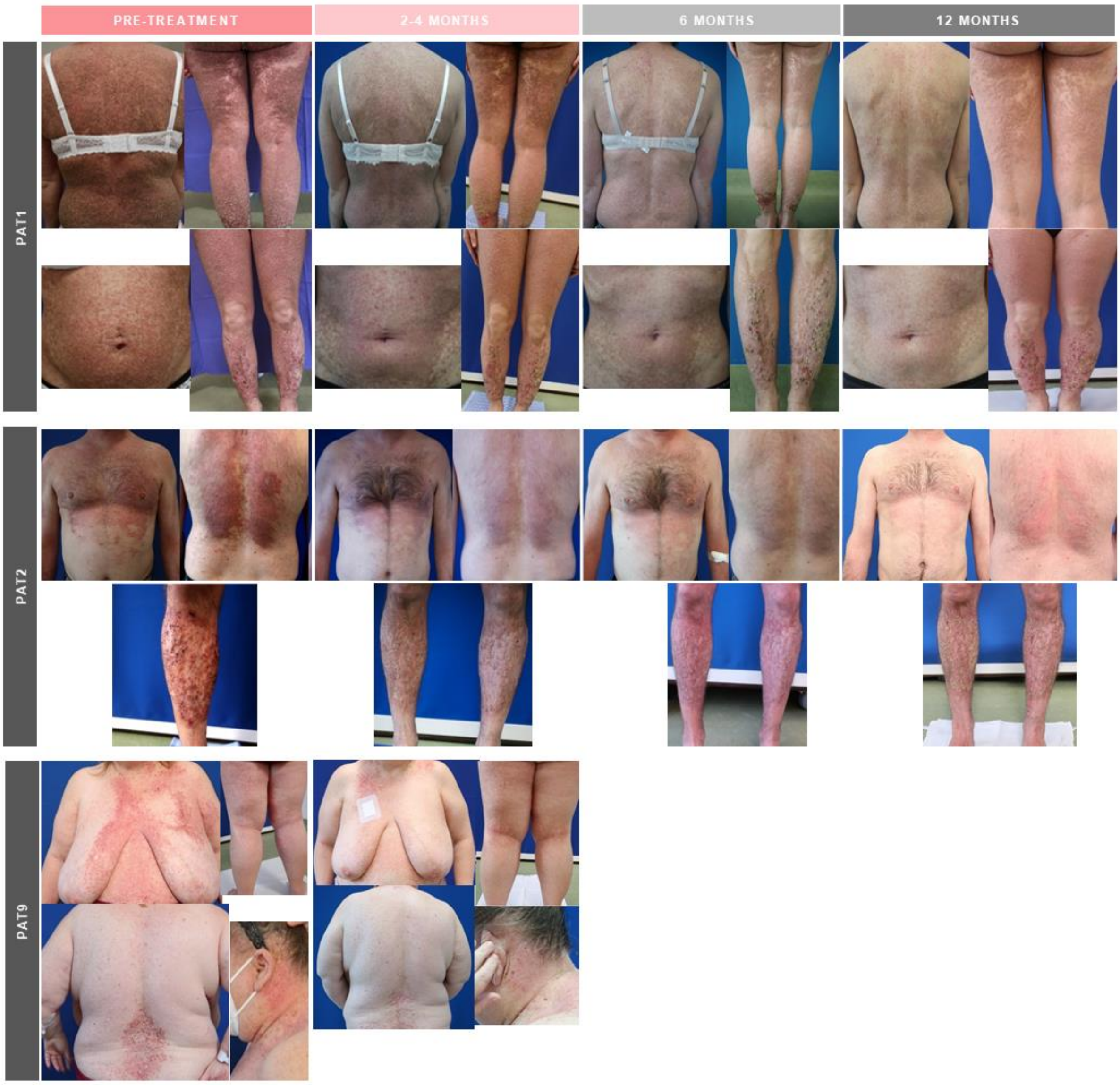

Supplementary Fig 8: Clinical pictures during treatment of DD PAT1, PAT2 and PAT9

Patient 1: Pre-treatment (prior to systemic therapy with guselkumab): erythematous and brownish hyperkeratotic papules and plaques on the entire integument, on the legs additionally crusts and erosions. 2 months post therapy: flattening of papules and plaques on the thorax, decrease of erythema. 6 months post therapy: further flattening of papules and plaques on the thorax. 12 months post treatment: flattened and reduced papules on the thorax, on the legs hyperkeratotic plaques and erythema, crusts and erosions.

Patient 2: Pre-treatment (before systemic therapy with secukinumab): erythematous and brownish hyperkeratotic papules and plaques on thorax and legs, on legs additionally crusts and erosions. 3 months post therapy: reduced erythema on the thorax, flattened papules on the thorax and plaques on the legs. 6 months post therapy: flattened papules on the thorax. 12 months post therapy: flattened and reduced papules on the thorax, hyperkeratotic plaques on the legs and lightened erythema.

Patient 9: Pre-treatment (prior to systemic therapy with secukinumab): erythematous and brownish hyperkeratotic papules and plaques on thorax, legs and face. 3.5 months post therapy: flattened plaques on thorax and legs.

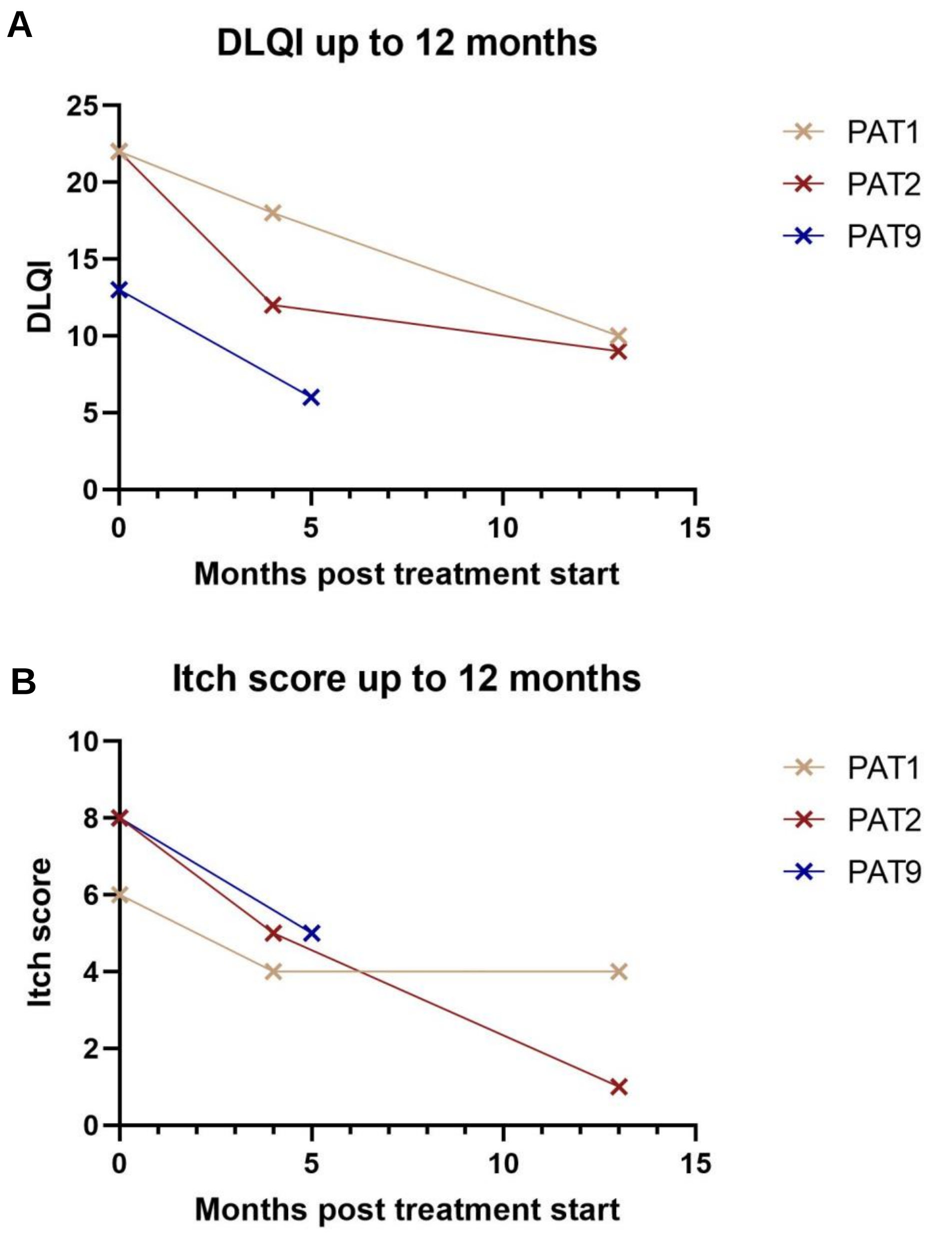

Supplementary Fig 9: Improved itch and quality of life of DD patients treated with biologics.

A Dermatology Life Quality Index questionnaire (DLQI) score of PAT1, PAT2 and PAT9. **B** Itch score (visual analogue scale 0-10) of PAT1, PAT2 and PAT9. Both scores were retrospectively collected from PAT1 and PAT2, while they were prospectively collected from PAT9.

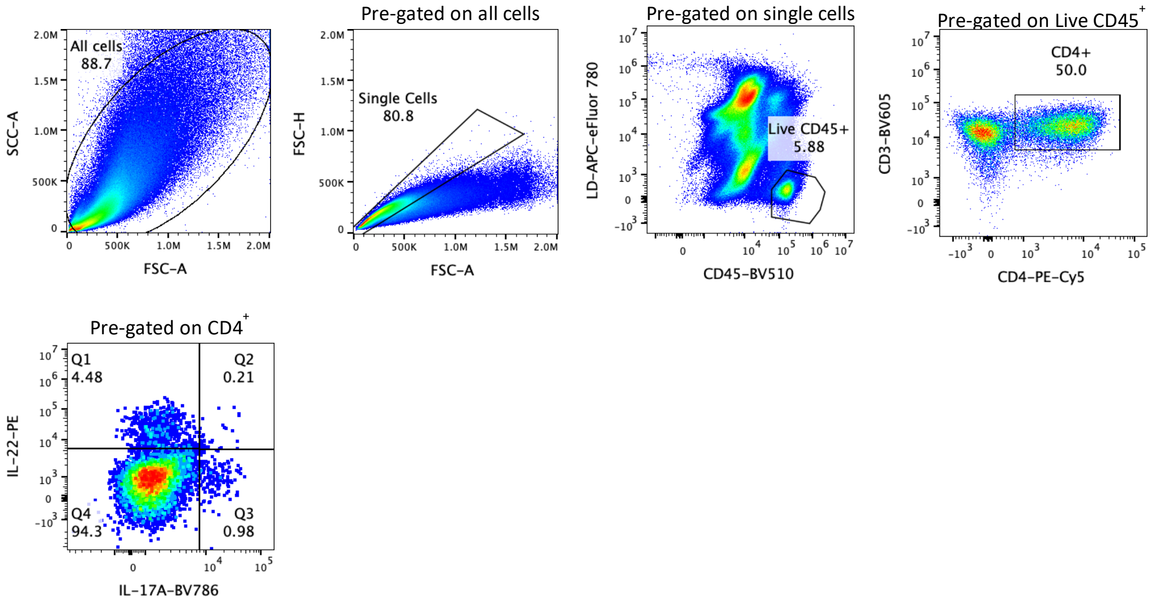

Supplementary Fig 10: Strategy for gating IL-17A$^+$ CD4$^+$ T cells in single cell suspension from human skin.

All cells were gated using physical parameter; forward scatter - area (FSC-A) and side scatter – area (SCC-A). Doublets were excluded using FSC-A and FSC-H. Further, dead cells were excluded (positive for APC eflour 780 signal) and live CD45$^+$ T cell population was gated. Thereafter, CD4$^+$ T cells were gated, which were further gated for IL-17A$^+$ and IL-22$^+$ CD4$^+$ T cells.

Supplementary Methods

Immunofluorescence.

Immunofluorescence was performed on formalin-fixed, paraffin-embedded lesional patient skin and normal control skin. After deparaffinization of the slides, Antigen Retrieval was performed in boiling hot citrate buffer (pH 6.0) for ten minutes. Slides were blocked, followed by overnight incubation of the 1:500 diluted IL-23 p19 Rabbit anti-Human Antibody (PA5-20239, Thermo Fisher Scientific), 1:200 diluted IL-17 Polyclonal Antibody (bs-2140R, Bioss) and 1:50 diluted Anti-Human CD3 Monoclonal Antibody (MA5-12577, Invitrogen). Antibodies Goat anti-Rabbit IgG (H+L) Cross-Adsorbed Secondary Antibody, AF 488 (A-11070, Thermo Fisher Scientific) and Donkey anti-Mouse IgG (H+L) Highly Cross-Adsorbed Secondary Antibody, AF 647 (A-31571, Invitrogen) were utilized as secondary antibodies. Slides were covered using Antifade/DAPI covering media and scanned with Histo Scanner VS200 v4 (Olympus).
