# Supplementary material for: Th17-associated cytokines IL-17 and IL-23 in inflamed skin of Darier disease patients as potential therapeutic targets
Source: Nat Commun. 2023 Nov 17;14:7470. doi: 10.1038/s41467-023-43210-5 (PMC10656568; doi:10.1038/s41467-023-43210-5)
Supplement: Supplementary file 3 — Reporting Summary [file 41467_2023_43210_MOESM3_ESM.pdf]

nature portfolio

Corresponding author(s): Wolfram Hötzenecker

Last updated by author(s): Oct 1, 2023

Reporting Summary

Nature Portfolio wishes to improve the reproducibility of the work that we publish. This form provides structure for consistency and transparency in reporting. For further information on Nature Portfolio policies, see our Editorial Policies and the Editorial Policy Checklist.

Statistics

For all statistical analyses, confirm that the following items are present in the figure legend, table legend, main text, or Methods section.

n/a	Confirmed	
☐	☒	The exact sample size (*n*) for each experimental group/condition, given as a discrete number and unit of measurement
☐	☒	A statement on whether measurements were taken from distinct samples or whether the same sample was measured repeatedly
☐	☒	The statistical test(s) used AND whether they are one- or two-sided<br>*Only common tests should be described solely by name; describe more complex techniques in the Methods section.*
☒	☐	A description of all covariates tested
☐	☒	A description of any assumptions or corrections, such as tests of normality and adjustment for multiple comparisons
☐	☒	A full description of the statistical parameters including central tendency (e.g. means) or other basic estimates (e.g. regression coefficient) AND variation (e.g. standard deviation) or associated estimates of uncertainty (e.g. confidence intervals)
☐	☒	For null hypothesis testing, the test statistic (e.g. *F*, *t*, *r*) with confidence intervals, effect sizes, degrees of freedom and *P* value noted<br>*Give P values as exact values whenever suitable.*
☒	☐	For Bayesian analysis, information on the choice of priors and Markov chain Monte Carlo settings
☒	☐	For hierarchical and complex designs, identification of the appropriate level for tests and full reporting of outcomes
☒	☐	Estimates of effect sizes (e.g. Cohen's *d*, Pearson's *r*), indicating how they were calculated

Our web collection on statistics for biologists contains articles on many of the points above.

Software and code

Policy information about availability of computer code

Data collection	Olympus VS200 ASW 3.2.a, Histech Pannormaic 3.0.3 - 250, CytExpert 2.4, LightCycler 480 Software 1.5.1.62, NanoString nCounter SPRINT
Data analysis	Nanostring nSolver 4.0, RStudio 2022.02.3+492, R version 4.3.1, Seurat v4.3.0.1, harmony v0.1.1, edgeR v3.42.4, CorelDraw 2021, GraphPad Prism 9.5.1 (733), GSEA 4.1.0, Microsoft Office Professional Plus 2019, Olympus OlyVIA 3.2.1, SlideViewer version 2.5QuPath 0.4.3, FlowJo LLC version 10

For manuscripts utilizing custom algorithms or software that are central to the research but not yet described in published literature, software must be made available to editors and reviewers. We strongly encourage code deposition in a community repository (e.g. GitHub). See the Nature Portfolio guidelines for submitting code & software for further information.

Data

Policy information about availability of data

All manuscripts must include a data availability statement. This statement should provide the following information, where applicable:

- Accession codes, unique identifiers, or web links for publicly available datasets
- A description of any restrictions on data availability
- For clinical datasets or third party data, please ensure that the statement adheres to our policy

The source data of this manuscript are provided as a Source Data file and are deposited in open public repositories. The Nanostring data generated in this study have ben deposited in the GEO database under accession code (GSE222043 [https://www.ncbi.nlm.nih.gov/geo/query/acc.cgi?acc=GSE222043]. The scRNAseq data of Darier's Disease patients generated in this study have been deposited in the GEO database under accession code , GSE235255 [https://www.ncbi.nlm.nih.gov/

geo/query/acc.cgi?acc=GSE235255]). The scRNA-seq of healthy and psoriasis data used is from publicly available dataset (GEO dataset GSE162183 [https://www.ncbi.nlm.nih.gov/geo/query/acc.cgi?acc=GSE162183]). The qPCR, FACS and quantification of IF/TMA generated for this manuscript are available in the Source Data file of the manuscript.

Research involving human participants, their data, or biological material

Policy information about studies with human participants or human data. See also policy information about sex, gender (identity/presentation), and sexual orientation and race, ethnicity and racism.

Reporting on sex and gender	All available DD patient samples were collected irrespective of sex or gender. Healthy controls were collected from both sexes. Psoriasis samples were collected according to availability, but trying to match the age group of the DD patients.<br>Sex was determined based on self-reporting. Three male and three female DD patients were included.
Reporting on race, ethnicity, or other socially relevant groupings	We did not collect any information on race, ethnicity or other socially relevant groupings.
Population characteristics	Samples were collected based on availability. All age groups and sexes were included. Age was collected in intervals of 5 years, ATP2A2 genotype was determined, disease severity was determined in terms of affected body surface area, prior complications and therapies, as well as current therapy are described in the manuscript.
Recruitment	All DD patients visiting our clinics and willing to participate in the study were included. We exluded patients with malignant co-morbidities from our study.<br>We treated only therapy refractory patients with a severe and pronounced phenotype and who were willing to receive a systemic treatment. Therefore, there might be a bias towards severe phenotype amongst treated patients.
Ethics oversight	Ethics committee of the Medical Faculty of Johannes Kepler University Linz and ethics committee of the University of Lausanne

Note that full information on the approval of the study protocol must also be provided in the manuscript.

Field-specific reporting

Please select the one below that is the best fit for your research. If you are not sure, read the appropriate sections before making your selection.

[x] Life sciences [ ] Behavioural & social sciences [ ] Ecological, evolutionary & environmental sciences

For a reference copy of the document with all sections, see nature.com/documents/nr-reporting-summary-flat.pdf

Life sciences study design

All studies must disclose on these points even when the disclosure is negative.

Sample size	Samples were collected based on availability. Three male and three female DD patients were included. We included as many as possible, but at least three (in order to be able to derive statistics) negative controls (healthy skin from dermatologic and plastic surgery) and positive controls (psoriasis patients).
Data exclusions	We excluded the data of DD PAT2 from the NanoString evaluation, as it did by far not pass quality control.
Replication	qPCR was performed at least twice for each patient (in tripilcate per run) and was reproducible for each patient.<br>NanoString was performed using only one sample per patient, as recommended in the manufacturer's protocol. As NanoString analysis was used to obtain a direction of immunologic response in DD patients, we did not replicate this experiment, but rather proofed its results using other mehtods (qPCR, IHC, Legendplex).<br>Due to sample availability (human skin biopsies), scRNA-Seq, FACS analysis and Legendplex analysis were performed once per patient or control.<br>IF and Opalstaining of TMA were performed at least twice per patient/TMA with similiar results. For TMA, different IL-17A antibodies were tested, with similiar results.
Randomization	Randomization was not applicable, as we included the patients based on their diagnosis, and all patients recieved treatment based on their skin immuno-profile.
Blinding	Blinding was not applicable, as we did not apply different drugs/placebos.

Reporting for specific materials, systems and methods

We require information from authors about some types of materials, experimental systems and methods used in many studies. Here, indicate whether each material, system or method listed is relevant to your study. If you are not sure if a list item applies to your research, read the appropriate section before selecting a response.

Materials & experimental systems

n/a	Involved in the study	
☐	☒	Antibodies
☒	☐	Eukaryotic cell lines
☒	☐	Palaeontology and archaeology
☒	☐	Animals and other organisms
☐	☒	Clinical data
☒	☐	Dual use research of concern
☒	☐	Plants

Methods

n/a	Involved in the study	
☒	☐	ChIP-seq
☐	☒	Flow cytometry
☒	☐	MRI-based neuroimaging

Antibodies

Antibodies used

L-23 p19 Rabbit anti-Human Antibody (PA5-20239, Thermo Fisher Scientific, polyclonal, LOT XB3495255)
IL-17 Polyclonal Antibody (bs-2140R, Bioss, polyclonal, LOT AO05136689)
Anti-Human CD3 Monoclonal Antibody (MA5-12577, Invitrogen, Clone F7.2.38, LOT CL3514017A)
Goat anti-Rabbit IgG (H+L) Cross-Adsorbed Secondary Antibody, AF 488 (A-11070, Thermo Fisher Scientific, polyclonal, LOT 4209258) and Donkey anti-Mouse IgG (H+L) Highly Cross-Adsorbed Secondary Antibody, AF 647 (A-31571, Invitrogen, polyclonal, LOT 2420713)
CD3-bv605 antibody (SK7; Biolegend, 344836, LOT B284954)
CD4-PE-Cy5 (RPY-T4; Biolegend, 300510, LOT B263820 and B295274)
CD45-bv510 (HI30; Biolegend, 304036, LOT B315939)
IL-17A-BV786 (N49-653; BD, 563745, LOT 0038820 and 1032263)
IL-22-PE (22URTI; eBioscience, 12-7229-42, LOT 2024855)
anti-CD3 PE-Cy5 (UCHT1, Biolegend, 300410, LOT B270168 and b354022)
anti-CD3 bv421 (UCHT1, Biolegend, 300434, LOT B285854 and B328663)
anti-CD4 PE/Dazzle594 (RPA-T4, Biolegend, 300548, LOT B284876)
anti-CD45RA AF700 (HI100, Biolegend, 304120, LOT B355316)
anti-CLA bv605 (HECA-452, BD, 563960, LOT 9018706)
anti-CD103 APC (BerACT8, Biolegend, 350216, LOT B332012)
anti-IL-17A PerCPcy5.5 (BL168, BioLegend, 512314, LOT B330530)
anti-CD4 (EPR6855, abcam, ab133616, LOT GR3276764-27)

Validation

L-23 p19 Rabbit anti-Human Antibody (PA5-20239, RRID:AB_11152389)
IL-17 Polyclonal Antibody (bs-2140R, RRID:AB_10855928)

CD3-bv605 antibody (SK7, RRID:AB_2565825)
CD4-PE-Cy5 (RPY-T4, RRID:AB_314078)
CD45-bv510 (HI30, RRID:AB_2561940)
IL-17A-BV786 (RN49-653, RID:AB_2738401)
IL-22-PE (22URTI, RRID:AB_1834463)

anti-CD3 PE-Cy5 (UCHT1, RRID:AB_314064)
anti-CD3 bv421 (UCHT1, RRID:AB_10962690)
anti-CD4 PE/Dazzle594 (RPA-T4, RRID:AB_2563566)
anti-CD45RA AF700 (HI100, RRID:AB_493763)
anti-CLA bv605 (HECA-452, RRID:AB_2738512)
anti-CD103 APC (BerACT8, RRID:AB_2563907)
anti-IL-17A PerCPcy5.5 (BL168, RRID:AB_961396)

anti-CD4 (EPR6855, RRID:AB_2750883)

Clinical data

Policy information about clinical studies

All manuscripts should comply with the ICMJEguidelines for publication of clinical research and a completedCONSORT checklist must be included with all submissions.

Clinical trial registration	NCT05680974
Study protocol	not applicable for our case series which can also be described as basic research withthree patients as proof of concept
Data collection	Data were collected at the Department of Dermatology, Kepler University Hospital Linz,at the outpatient clinic and ward hospital. Patients were recruited from the genodermatosis consultation hour, who presented either planned at the outpatient unit or at the ward with a deterioration of the skin condition. Periods of time for recruitment and data were collected between 03/22-03/23.
Outcomes	Primary outcome: Improvement of skin manifestations as assessed by IGA score, DLQI and itchscore upon off-label treatment with approved monoclonal antibody targeting the overexpressed cytokine in inflamed lesional skin.

Flow Cytometry

Plots

Confirm that:

- [x] The axis labels state the marker and fluorochrome used (e.g. CD4-FITC).
- [x] The axis scales are clearly visible. Include numbers along axes only for bottom left plot of group (a 'group' is an analysis of identical markers).
- [x] All plots are contour plots with outliers or pseudocolor plots.
- [x] A numerical value for number of cells or percentage (with statistics) is provided.

Methodology

Sample preparation	For skin flow cytometry staining, biopsies were thawed, weighed and minced into smaller pieces using surgical scissors and were digested overnight with 1 ml skin digestion mix containing collagenase type 4 (0.8mg/ml; Worthington, LS004186) and DNAse (11.77 Units/ml; Sigma-Aldrich, D5319) in RPMI-complete (RPMIc) for about 0.1 g of skin at 37°C in 5% CO2 incubator (RPMIc: RPMI 1640 (Gibco, 31870074) with 5% human serum (One lambda, A25761), 1% penicillin/streptomycin (Sigma-Aldrich, P0781), 1% l-glutamine (Gibco, A2916801), 1% non-essential amino acid solution (NEAA) (Gibco, 11140035), 1% sodium pyruvate (Sigma-Aldrich, S8636), and 0.1% β-mercaptoethanol (Gibco, 31350010)). After overnight digestion, the cell suspension was filtered through 70 µm mesh, then washed and resuspended in RPMIc. For detection of intracellular cytokines, skin single-cell suspensions were stimulated with phorbol 12-myristate 13-acetate (PMA) (50 ng/ml; Sigma-Aldrich, P8139) and ionomycin (1 µg/ml; Sigma-Aldrich, I06434) with brefeldin A (10 µg/ml; Sigma-Aldrich, B6542) for 3 hours at 37 °C in 5% CO2 incubator. Cells were incubated for 15mins in Fc receptor blocking solution (BioLegend Cat# 422302, RRID:AB_2818986). Thereafter, cells were stained in PBS containing the fixable viability dye eFluor® 780, (Thermo Fisher Scientific Cat# 65-0865-14) for surface markers, using antibodies: anti-CD3 bv605, (BioLegend Cat# 344836, RRID:AB_2565825); anti-CD4 PE-Cy5, (BioLegend Cat# 300510, RRID:AB_314078); anti-CD45 bv510, (BioLegend Cat# 304036, RRID:AB_2561940) and fixed and permeabilized using Foxp3 staining kit (Thermo Fisher Scientific Cat# 00-5523-00) for staining the intracellular markers using antibodies: anti-IL-17A BV786, (BD Biosciences Cat# 563745, RRID:AB_2738401); anti-IL-22 PE (22URTI), (Thermo Fisher Scientific Cat# 12-7229-42, RRID:AB_1834463). After stimulation all the steps were done at 4°C.<br>For PBMCs flow cytometry, cryopreserved PBMCs were thawed and rested overnight in RPMIc at 37°C in 5% CO2 incubator. Cells were then stimulated with PMA/ionocycin/brefeldin A followed by Fc receptor blocking and stained in PBS containing viability dye as described above for surface markers, using antibodies: anti-CD3 PE-Cy5, (BioLegend Cat# 300410, RRID:AB_314064); anti-CD3 bv421, (BioLegend Cat# 300434, RRID:AB_10962690); anti-CD4 PE/Dazzle594, (BioLegend Cat# 300548, RRID:AB_2563566) ; anti-CD4 PE-Cy5, (BioLegend Cat# 300510, RRID:AB_314078); anti-CD45RA AF700, (BioLegend Cat# 304120, RRID:AB_493763); anti-CLA bv605, (BD Biosciences Cat# 563960, RRID:AB_2738512); anti-CD103 APC, (BioLegend Cat# 350216, RRID:AB_2563907). Cells were fixed and permeabilized using Cytofix/Cytoperm kit (BD Biosciences Cat# RUO 554714) for detection of intracellular IL-17A cytokine, using antibody: anti-IL-17A PerCPcy5.5, (BioLegend Cat# 512314, RRID:AB_961396) .
Instrument	Cytoflex LS (Beckman Coulter) flow cytometer
Software	CytExpert 2.4 software, FlowJo software (FlowJo LLC, version10), GraphPad prism (version 9)
Cell population abundance	We did not perform any sorting experiment.
Gating strategy	Gating strategy for IL-17A+ CD4+ cTrm in PBMCs: Lymphocytes were gated using physical parameters; forward scatter - area (FSC-A) and side scatter – area (SCC-A). Doublets were excluded using FSC-A and FSC-H. Further, dead cells were excluded (positive for APC eflour 780 signal) and live CD3+ T cell population was gated. Thereafter, CD4+ T cells were gated for memory cTrm population as CD45RA-CLA+CD103+ and then finally for IL17A producing population.<br>Gating strategy for IL-17A+ CD4+ T cells in single cell suspension from human skin: All cells were gated using physical parameter; forward scatter - area (FSC-A) and side scatter – area (SCC-A). Doublets were excluded using FSC-A and FSC-H. Further, dead cells were excluded (positive for APC eflour 780 signal) and live CD45+ T cell population was gated. Thereafter,

CD4+ T cells were gated, which were further gated for IL-17A+ and IL-22+ CD4+ T cells.

☒ Tick this box to confirm that a figure exemplifying the gating strategy is provided in the Supplementary Information.
